# Distinct Dynamics of Striatal and Prefrontal Neural Activity During Temporal Discrimination

**DOI:** 10.3389/fnint.2018.00034

**Published:** 2018-08-13

**Authors:** Jieun Kim, Dohoung Kim, Min Whan Jung

**Affiliations:** ^1^Center for Synaptic Brain Dysfunctions, Institute for Basic Science (IBS), Daejeon, South Korea; ^2^Graduate School of Medical Science and Engineering, Korea Advanced Institute of Science and Technology (KAIST), Daejeon, South Korea; ^3^Department of Biological Sciences, Korea Advanced Institute of Science and Technology (KAIST), Daejeon, South Korea

**Keywords:** interval timing, temporal categorization task, prefrontal cortex, striatum, rats

## Abstract

The frontal cortex-basal ganglia circuit plays an important role in interval timing. We examined neuronal discharges in the dorsomedial and dorsolateral striatum (DMS and DLS) in rats performing a temporal categorization task and compared them with previously recorded neuronal activity in the medial prefrontal cortex (mPFC). All three structures conveyed significant temporal information, but striatal neurons seldom showed the prolonged, full-interval spanning ramping activity frequently observed in the mPFC. Instead, the majority fired briefly during sample intervals. Also, the precision of neural time decoding became progressively worse with increasing time duration in the mPFC, but not in the striatum. With the caveat that mPFC and striatal units were recorded from different animals, our results suggest that the striatum and mPFC convey temporal information via distinct neural processes.

## Introduction

The frontal cortex-basal ganglia circuit has been strongly implicated in interval timing—the estimation of time intervals in the range of seconds to minutes (Buhusi and Meck, 2005; Meck et al., 2008). Brain imaging studies in humans have found enhanced blood-oxygen-level dependent signals in the frontal cortex and the striatum during various timing tasks (Rao et al., 1997; Lewis and Miall, 2003; Nenadic et al., 2003; Coull et al., 2004; Hinton and Meck, 2004; Jahanshahi et al., 2006; Grahn and Brett, 2007; Penney and Vaitilingam, 2008; Grahn and McAuley, 2009; Koch et al., 2009; Teki et al., 2011; Geiser et al., 2012). Local lesions or inactivation of the prefrontal cortex (PFC; Glickstein et al., 1964; Dietrich et al., 1997; Dietrich and Allen, 1998; Mangels et al., 1998; Onoe et al., 2001; Koch et al., 2003; Jones et al., 2004; Kim et al., 2009) or striatum (Artieda et al., 1992; Meck, 1996, 2006; Gibbon et al., 1997; Rammsayer and Classen, 1997; Harrington et al., 1998; Harrington and Haaland, 1999; Chiba et al., 2015; Gouvêa et al., 2015) impair interval timing behavior in both humans and animals. Physiological studies have shown temporal changes in neuronal activity in the PFC, premotor cortex and striatum in monkeys and rats performing various interval timing tasks (Brody et al., 2003; Matell et al., 2003; Roux et al., 2003; Reutimann et al., 2004; Sakurai et al., 2004; Genovesio et al., 2006, 2009; Oshio et al., 2006; Tanaka, 2007; Chiba et al., 2008, 2015; Lebedev et al., 2008; Jin et al., 2009; Mita et al., 2009; Kim et al., 2013; Knudsen et al., 2014; Gouvêa et al., 2015; Mello et al., 2015; Murakami et al., 2017; Mendoza et al., 2018). In addition, optogenetic stimulation of D1 receptor-expressing neurons in the PFC alters interval timing behavior in mice (Narayanan et al., 2012).

Although a large body of evidence indicates the involvement of the frontal cortex and basal ganglia in interval timing, their respective roles in interval timing and underlying neural processes require further study. To this end, we sought to compare timing-related neural activity between connected regions of the frontal cortex and basal ganglia under the same behavioral setting. We showed previously that in rats performing a temporal categorization task, some medial PFC (mPFC) neurons convey temporal information in the form of monotonically changing (ramping) activity. In these rats, the activity of the recorded mPFC neuronal ensemble was tightly correlated with time interval discrimination behavior (Kim et al., 2013). Here, to better understand how the frontal cortex-basal ganglia circuit contributes to interval timing, we examined neuronal activity in the striatum in the same temporal categorization task we used in our previous study (Kim et al., 2013) so we could compare the results with our results in the mPFC.

The cortex-basal ganglia circuit is thought to consist of multiple parallel loops (Alexander et al., 1986; Alexander and Crutcher, 1990; Graybiel, 2008; Redgrave et al., 2010). In rats, the dorsomedial and dorsolateral striatum (DMS and DLS, respectively) appear to be parts of distinct cortico-basal ganglia loops; they have distinct anatomical connection patterns (McGeorge and Faull, 1989; Voorn et al., 2004; Balleine et al., 2007; Redgrave et al., 2010; Devan et al., 2011) and their inactivation or lesions yield dissociable changes in the animal’s choice behavior (Yin et al., 2004, 2005; Balleine et al., 2007; White, 2009). The DMS receives direct projections from the mPFC and is part of the associative cortico-basal ganglia loop, while the DLS receives direct projections from the sensorimotor cortex and is part of the sensorimotor cortico-basal ganglia loop (McGeorge and Faull, 1989; Voorn et al., 2004; Balleine et al., 2007; Redgrave et al., 2010; Devan et al., 2011). We recorded neuronal discharges from both the DMS and DLS and compared them to neuronal discharges recorded from the mPFC. We found that both striatal and mPFC neurons carry information about elapsed time, but timing-related neural activity dynamics differ between the two regions.

## Materials and Methods

### Subjects

Three young male Sprague-Dawley rats (approximately 9–11 weeks old; 280–380 g) were used. After 1 week of extensive handling and water deprivation, their body weights fell to 80%–85% of their *ad libitum* weights. Once behavioral training began, they were restricted to 30 min of water access immediately after finishing their once-daily behavioral session. Experiments were performed in the dark phase of a 12 h light/dark cycle. All animal care and experimental procedures were performed in accordance with protocols approved by the directives of the Animal Care and Use Committee of the Korea Advanced Institute of Science and Technology (Daejeon, South Korea).

### Behavioral Task

**Figure 1:**
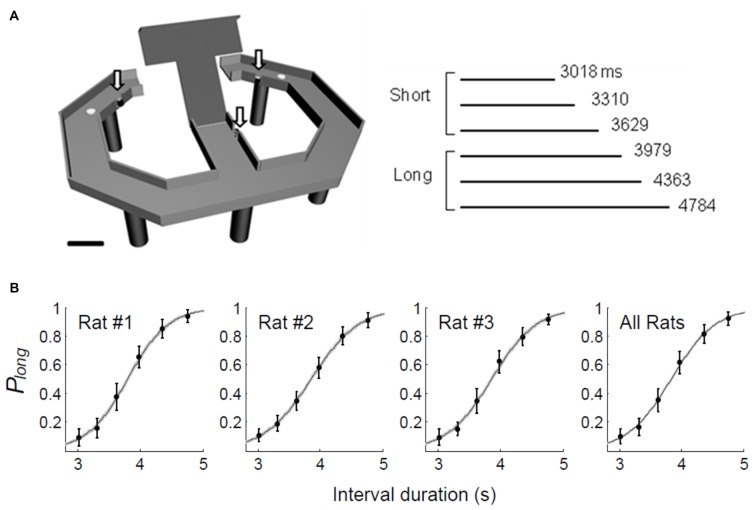
Behavioral performance. **(A)** The rats performed a temporal categorization task in a modified T-maze. They were rewarded when they discriminated six sample intervals into short or long and visited the corresponding targets (white circles). Arrows, photobeam sensors. Calibration, 10 cm. **(B)** The fraction of long-target choice (*P*_long_) as a function of sample interval duration. The solid lines were determined by logistic regression and the shading indicates the 95% confidence interval. Error bars, SD.

We used the same temporal categorization task described in our previous studies (Kim et al., 2009, 2013). Briefly, the rats were required to discriminate six randomly presented sample intervals into short (3,018, 3,310 and 3,629 ms) or long (3,979, 4,363 and 4,784 ms) and navigate to the corresponding target sites (long-choice target, left in one rat and right in two rats) to obtain a 30 μl water reward (Figure 1A). The animals were required to come back from the target sites to the central stem via lateral alleys. The onset of a sample interval was triggered by the animal’s breaking of the central photobeam. The onset of the sample interval was accompanied by a brief tone (200 ms, 3.3 KHz, 90 dB) and its offset was marked by the lowering of the central bridge. Each session consisted of an average of 250.7 trials (SD 14.1). The animals were over-trained before unit recordings (30–49 and 14–24 days of training before and after electrode implantation, respectively). They also experienced 10 practice trials, five with the shortest interval and five with the longest interval, before each recording session.

### Unit Recordings

**Figure 2:**
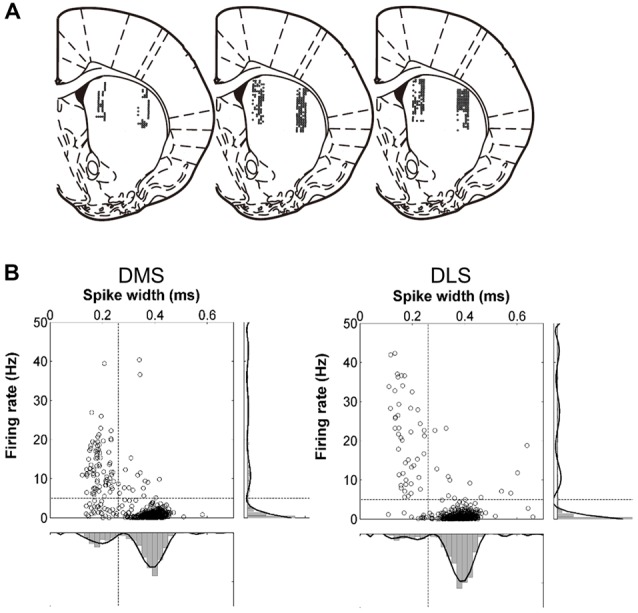
Recording sites and unit classification. **(A)** Single units were recorded simultaneously from the dorsomedial striatum (DMS) and dorsolateral striatum (DLS). The diagrams are coronal section views of three rat brains 0.48 mm posterior to Bregma. Each diagram represents one rat and each circle represents one recording site that was determined based on histology and electrode advancement history. Modified with permission from Paxinos and Watson (1998). **(B)** Unit classification. Recorded units were classified into putative medium spiny neurons (MSNs) and putative interneurons based on mean discharge rates and filtered spike waveform widths. The black lines on the right and at the bottom indicate 8th-order polynomial fits. Those units with mean firing rates <5 Hz and spike widths >0.26 ms were classified as putative MSNs and the rest were classified as putative interneurons.

Two sets of six tetrodes were implanted in the DMS (centered 0.5 mm anterior and 1.8 mm lateral to Bregma; 3.4–5.9 mm ventral to the brain surface) and DLS (centered 0.5 mm anterior and 3.8 mm lateral to Bregma; 3.4–5.9 mm ventral to the brain surface) of well-trained rats under deep anesthesia with sodium pentobarbital (50 mg/kg; Figure 2A). After >1 week of recovery from surgery, the tetrodes were gradually lowered to obtain isolated unit signals. Once unit recording began, the tetrodes were advanced by 100 μm after each daily recording session. Unit signals were amplified 10,000×, filtered between 600 and 6,000 Hz, digitized at 32 kHz, and stored on a personal computer using the Cheetah data acquisition system (Neuralynx, Bozeman, MT, USA). The animal’s head position was monitored by tracking at 60 Hz a set of light-emitting diodes mounted on the headstage. Spike clustering was performed offline using MClust (A.D. Redish). Only well-isolated unit clusters were included in the analysis (L ratio <0.2, isolation distance >15). After completing the recordings, small marking lesions were made by passing an electrolytic current (50 mA, 30 s, cathodal) through one channel of each tetrode. Then, the recording locations were verified histologically as previously described (Baeg et al., 2001).

### Analysis

#### Choice Behavior

Animal choice data were subjected to the following logistic regression analysis:

(1) $$\text{log}\left(\frac{P_{\text{long}}}{1-P_{\text{long}}}\right)=a+bT,$$

where *P*_long_ is the probability to choose the long target (averaged across all sessions for each animal and each sample duration), *T* is the sample duration, and *a* and *b* are constants.

#### Unit Classification

Units with mean firing rates <5 Hz and spike widths >0.26 ms were classified as putative medium spiny neurons (MSNs) and the rest were classified as putative interneurons (Figure 2B).

#### Activity Duration

A spike density function was generated for each neuron for the longest sample interval (4784 ms) by applying a Gaussian kernel to each spike (σ = 100 ms). Activity half-duration was defined as the duration between the maximal and half-maximal activity of the spike density function. When a spike density function yielded two half-durations, the longer one was used to calculate the activity half-duration for that neuron.

#### Ramping Activity

The longest sample interval was divided into four equal-duration bins for each neuron. If neural firing rates of the four bins varied significantly (one-way ANOVA, *p* < 0.05) and neural firing rates changed monotonically across the four bins, the neuron was considered as showing full-interval spanning ramping activity.

#### Multiple Linear Regression Analysis

To determine whether linear or logarithmic functions better explain individual neuronal activity, we divided each sample interval into 10 equal bins. The relationship between the mean firing rate of a neuron within each time bin and the time since interval onset was described by the following equation:

(2) $$S=a_{0}+a_{1}T+a_{2}PC+a_{3}X+a_{4}Y+a_{\text{5}}D+\varepsilon ,$$

where *S* is the trial-by-trial firing rate of a single neuron within a specific time bin, *T* is the raw or log-transformed time since the interval onset, *X*, *Y* and *D* indicate the animal’s mean lateral head position (*X*-position), mean vertical head position (*Y*-position), and overall displacement, respectively, in the corresponding analysis time window, *PC* denotes the animal’s goal choice in the previous trial, ε is the error term, and *a*_0_–*a*_5_ are regression coefficients.

#### Neural Decoding of Temporal Information

Neural decoding of temporal information was done as described previously (Kim et al., 2013). Briefly, we examined how well neuronal ensemble activity (simultaneously recorded neurons or all neurons pooled across sessions) during the last 500 ms of each sample interval classified sample intervals as short or long using a linear discriminant analysis. When analyzing error trials, we excluded the shortest and longest sample intervals from the analysis because of low error rates (9.7% and 7.9%, respectively). We removed a single trial and generated a linear discriminant function based on the neuronal ensemble activity in the remaining correct trials separated according to the correct target (short vs. long). We repeated this procedure for all correct and error trials and calculated the rate at which the model correctly classified sample interval lengths. The numbers of correct and error trials were equalized across sessions for each sample interval when decoding temporal information using all neurons pooled across sessions.

To assess the amount of temporal information conveyed by neuronal ensemble activity after controlling for the influence of movement-related variables, we used the partial residuals (ε_1_, Larsen and McCleary, 1972) of the following regression model for the classification of sample interval lengths:

(3) $$\varepsilon_{1}=S-\left(a_{2}PC+a_{3}X+a_{4}Y+a_{5}D\right),$$

where *S, X*, *Y* and *D* are neuronal firing rate, the animal’s mean *X*-position, mean *Y*-position and overall displacement, respectively, during the last 500 ms of each sample interval.

#### Statistical Analysis

Student’s *t*-tests were used to test the statistical significance of regression coefficients. The Kruskal-Wallis test was used to compare the activity durations of DMS, DLS and mPFC neurons. One-way ANOVA was used to compare the decoding performances of DMS, DLS and mPFC neurons. Two-tailed tests were employed for all statistical tests. A *p*-value < 0.05 was used as the criterion for a significant statistical difference. Data are expressed as means and SD.

## Results

### Behavior

Across all trials and all animals, we observed a 79.0% (SD 1.4) success rate in choosing the correct target. The probability of choosing the long interval target increased as a function of sample interval across all animals. A logistic regression model accounts well for the relationship between the probability of long target choice and interval duration (eq. 1; animal #1, *R*^2^ = 0.958; animal #2, *R*^2^ = 0.965; animal #3, *R*^2^ = 0.959; Figure 1B).

### Neuronal Database

We obtained a total of 490 well-isolated units from the DMS and 577 from the DLS in 71 recording sessions (Figure 2A). In the DMS, 355 (72.4%) were classified as putative MSNs and 135 (27.6%) were classified as putative interneurons; in the DLS, 485 (84.1%) were classified as putative MSNs and 92 (15.9%) were classified as putative interneurons (Figure 2B). The mean discharge rates of the putative MSNs were 0.88 Hz (SD 0.90) in the DMS and 0.73 Hz (SD 0.80) in the DLS. The mean discharge rates of the putative interneurons were 10.82 Hz (SD 8.98) in the DMS and 15.14 Hz (SD 14.99) in the DLS. For comparison, we re-analyzed recordings of 993 well-isolated units from the mPFC of rats performing the same task (Kim et al., 2013). Of these, 791 putative pyramidal cells had a mean discharge rate of 3.30 Hz (SD 0.13) and 202 putative interneurons had a mean discharge rate of 12.12 Hz (SD 0.69). We included in the analysis only putative MSNs and putative pyramidal cells with mean discharge rates >0.1 Hz during the task (DMS, *n* = 311, 1.00 Hz (SD 0.90); DLS, *n* = 429, 0.82 Hz (SD 0.81); mPFC, *n* = 782, 3.34 Hz (SD 3.76)). We failed to find significant variations in timing-related striatal neural activity along the dorsoventral axis except activity duration (see below).

### Activity Duration

**Figure 3:**
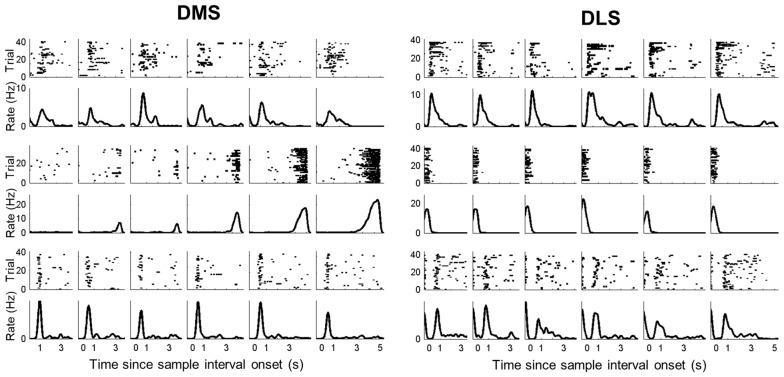
Examples of striatal neuronal activity during sample presentation. Spike raster plots and spike density functions (σ = 100 ms) of three DMS (left) and three DLS (right) MSNs. Trials were grouped according to sample interval length (left to right, short to long). The vertical lines indicate the onset of each time interval.

**Figure 4:**
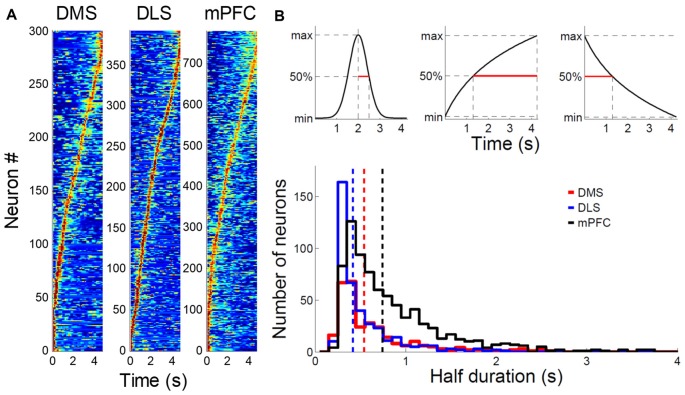
Comparison of striatal and medial prefrontal cortex (mPFC) neuronal activity profiles. **(A)** Normalized activity profiles of putative MSNs recorded from the DMS and DLS and putative pyramidal cells recorded from the mPFC (Kim et al., 2013) are shown for the longest sample interval (4,784 ms). Units were aligned according to the time of peak firing (red). Dark blue indicates no firing. **(B)** Top, activity half-duration was defined as the duration between the maximal and half-maximal activity for each neuron. Bottom, activity half-duration was estimated for putative MSNs of the DMS (red) and DLS (blue) as well as putative pyramidal cells of the mPFC (black) for the longest sample interval. Dashed lines denote mean half-durations in corresponding colors. Only correct trials were included in the analysis.

In a large proportion of putative MSNs recorded from the DMS and DLS, we observed phasic activity patterns at different times during the sample intervals (examples shown in Figure 3). Figure 4A shows activity profiles during the longest sample interval (4,784 ms) for all the striatal MSNs and mPFC pyramidal neurons we analyzed. As shown, the DLS and DMS MSNs tended toward phasic discharges, with the DLS neurons showing more brief discharges. The putative pyramidal cells in the mPFC tended toward broader temporal activity profiles, with some showing prolonged ramping (i.e., gradually increasing or decreasing during the entire sample interval). To quantify the activity duration for each individual neuron during the longest sample interval, we measured the length of time between the maximum and half-maximum level of each neuron’s spike density function and plotted it as the “Activity half-duration” in Figure 4B. The mean activity half-duration was the longest for the mPFC neurons and shortest for the DLS neurons (DMS, 542.5 ms (SD 477.1); DLS, 417.6 ms (SD 348.9); mPFC, 744.9 ms (SD 578.5); Kruskal-Wallis test, *F*_(2,1472)_ = 178.84, *p* < 0.001; Tukey-Kramer *post hoc* test, DMS vs. DLS, *p* < 0.001; DMS vs. mPFC, *p* < 0.001; DLS vs. mPFC, *p* < 0.001; effect size, *η*^2^ = 0.108; Figure 4B). Also, the proportions of neurons showing full-interval spanning ramping activity (see “Materials and Methods” section) were 25.8, 16.5 and 12.6% in the mPFC, DMS and DLS, respectively, which deviated significantly from an equal distribution (*χ*^2^-test, *χ*^2^ = 31.5; *p* < 0.001; Mann-Whitney *post hoc* test with Bonferroni correction, DMS vs. DLS, *p* = 0.146; DMS vs. mPFC, *p* = 0.001; DLS vs. mPFC, *p* < 0.001).

To test whether the activity duration varies along the dorsoventral axis of the striatum, we divided the DMS and DLS units into tertiles according to their recording depths and compared their activity durations. More ventral units tended to show shorter activity half-durations in the DMS (dorsal, 605.0 ms (SD 495.7); middle, 580.0 ms (SD 536.5); ventral, 445.0 ms (SD 373.7); Kruskal-Wallis test, *F*_(2,297)_ = 7.94, *p* = 0.019; Tukey-Kramer *post hoc* test, dorsal vs. middle, *p* = 0.594; dorsal vs. ventral, *p* = 0.015; middle vs. ventral, *p* = 0.168) as well as DLS (dorsal, 474.0 ms (SD 362.8); middle, 429.8 ms (SD 393.2); ventral, 348.9 ms (SD 269.0); *p* = 0.001; dorsal vs. middle, *p* = 0.096; dorsal vs. ventral, *p* < 0.001; middle vs. ventral, *p* = 0.239).

### Temporal Information

**Figure 5:**
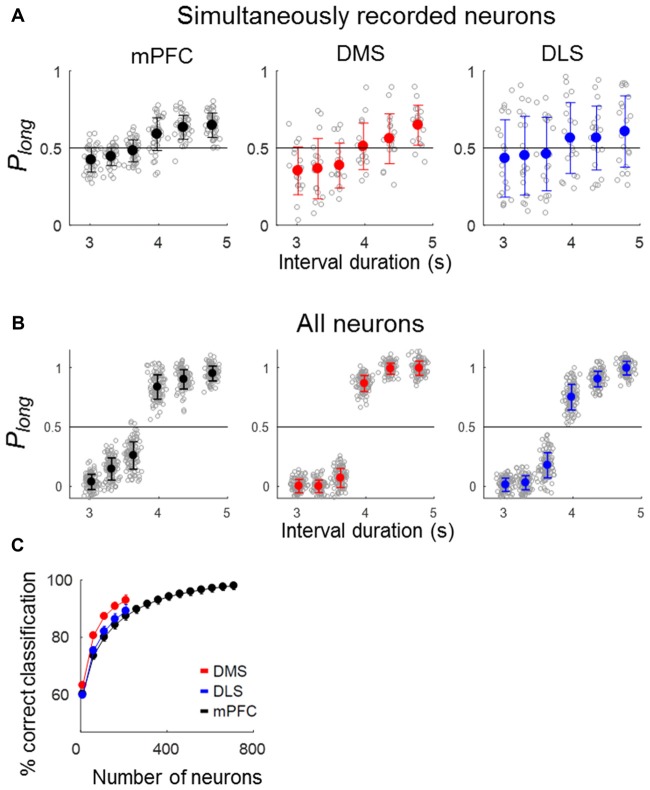
Neural classification of sample-interval length. Sample intervals were classified as short or long based on neuronal ensemble activity during the last 500 ms of each sample interval using a discriminant analysis. Only correct trials were included in the analysis. **(A)** Classification of sample-interval length based on simultaneously recorded units. Neural classification was performed using six neurons that were selected randomly from each simultaneously recorded ensemble, and this was repeated 100 times. Each gray circle denotes the outcome of 100 classifications obtained from each ensemble. Circles with saturated colors (black, blue and red) denote their means. *P*_long_, the fraction of long-sample classification. **(B)** Classification of sample-interval length based on all recorded units pooled across sessions (ensemble size = 200 neurons). The same as in **(A)** except that all recorded neurons were included in the analysis. **(C)** Results of a neuron dropping analysis applied to all recorded units. Shown are outcomes of neural classification as a function of ensemble size in steps of 50 neurons starting from an ensemble size of 10 neurons. A given number of units were selected randomly for each ensemble size, and this was repeated 100 times. Error bars, SD.

To quantify the temporal information carried by a neuronal population, we asked how well neuronal ensemble activity during the last 500 ms of each sample interval classifies sample intervals into short and long ones using correct trials. Neural classification of sample-interval length based on simultaneously recorded units was well above chance level (50%) in all areas (ensemble size = 6 neurons, mPFC, 59.0% (SD 0.76) correct classification; *t*-test, *p* < 0.001; DMS, 61.4% (1.1), *p* < 0.001; DLS, 56.9% (SD 1.2), *p* < 0.001; Figure 5A). Performance increased when the length classification was based on all units pooled across sessions (ensemble size = 200 neurons, mPFC, 88.8% (SD 3.47), *p* < 0.001; DMS, 97.4% (SD 0.92), *p* < 0.001; DLS, 92.4% (SD 2.42), *p* < 0.001; Figure 5B). We then performed a neuron-dropping analysis to examine the relationship between ensemble size and temporal information. At equivalent ensemble sizes, DMS ensembles conveyed significantly more temporal information than the DLS and mPFC ensembles (one-way ANOVA, ensemble size = 100 neurons, *F*_(2,297)_ = 1741.83, *p* < 0.001, Tukey’s HSD *post hoc* test, DMS vs. DLS, *p* < 0.001; DMS vs. mPFC, *p* < 0.001; ensemble size = 200 neurons, *F*_(2,297)_ = 512.01, *p* < 0.001, DMS vs. DLS, *p* < 0.001; DMS vs. mPFC, *p* < 0.001; Figure 5C).

**Figure 6:**
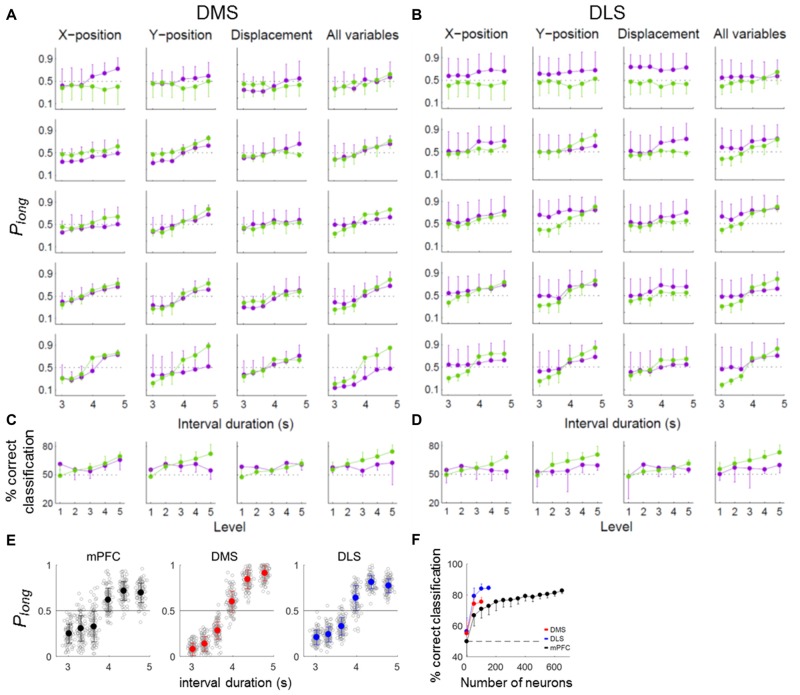
Effect of behavior on timing-related neural activity. **(A–D)** Neural coding of temporal information for different levels of behavioral variation. To determine whether the temporal information conveyed by DMS and DLS neural activity could be attributed to variations in the animal’s ongoing behavior during each sample interval, we compared neural decoding performance across behavioral sessions by dividing them into quintiles according to the amount of temporal information conveyed by each of the three behavioral variables (*X*-position, *Y*-position and displacement) or by all three together. Purple, neural decoding results. Green, behavioral decoding results. **(A,B)** Probability of long classification (*P*_long_) as a function of sample interval duration. **(C,D)** Mean % correct classification as a function of behavioral variation. **(E,F)** Classification of sample-interval length using partial residuals. **(E)** Length classification was done as in Figure 5B except thatthe partial residuals of a linear regression model containing the behavioral variables (*X*-position, *Y*-position and displacement) were used for the linear discriminant analysis instead of raw firing rates. Only correct trials were analyzed. **(F)** Results of a neuron dropping analysis. The same format as in Figure 5C. Error bars, SD.

We have shown previously that timing-related mPFC neural activity in the current task cannot be accounted for by behavioral variations during sample presentation (Kim et al., 2013). We performed the same analysis for the striatal neurons. For this, we divided the behavioral sessions into quintiles according to temporal information carried by each or whole set of three behavioral variables (the animal’s *X*-position, *Y*-position and displacement) and repeated the neural decoding analysis for each group. These behavioral variables did convey temporal information in varying degrees (Figures 6A,B). However, the pattern of neural decoding was generally similar across the quintiles (Figures 6A,B) so that there was no significant variation in the percentage of correct neural classification across the quintiles (one-way ANOVA, DMS, *X*-position, *F*_(4,25)_ = 1.94, *p* = 0.135; *Y*-position, *F*_(4,25)_ = 1.38, *p* = 0.268; displacement, *F*_(4,25)_ = 0.92, *p* = 0.469; all variables, *F*_(4,25)_ = 0.47, *p* = 0.758; DLS, *X*-position, *F*_(4,25)_ = 0.23, *p* = 0.919; *Y*-position, *F*_(4,25)_ = 0.48, *p* = 0.751; displacement, *F*_(4,25)_ = 0.70, *p* = 0.596; all variables, *F*_(4,25)_ = 0.46, *p* = 0.767; Figures 6C,D). Thus, neural decoding did not vary significantly as a function of behavioral decoding.

To further address this matter, we used the partial residuals (Larsen and McCleary, 1972) of the regression model containing the movement-related variables (*X*-position, *Y*-position and displacement; eq. 3) instead of raw firing rates for the length classification. All three areas conveyed significant amounts of temporal information, although their performances were somewhat lower (asymptotic values, ~80% correct classification; Figures 6E,F) compared to those using raw firing rates (asymptotic values, ~90% correct classification; Figure 5C). At equivalent ensemble sizes, the DMS and DLS ensembles conveyed significantly more temporal information than the mPFC ensemble (one-way ANOVA, ensemble size = 100 neurons, *F*_(2,297)_ = 286.05, *p* < 0.001, Tukey’s HSD *post hoc* test, DMS vs. DLS, *p* < 0.001; DMS vs. mPFC, *p* < 0.001; DLS vs. mPFC, *p* < 0.001). These results indicate relatively minor contributions of behavioral variations to the temporal information conveyed by striatal neural activity.

### Error Trial Analysis

**Figure 7:**
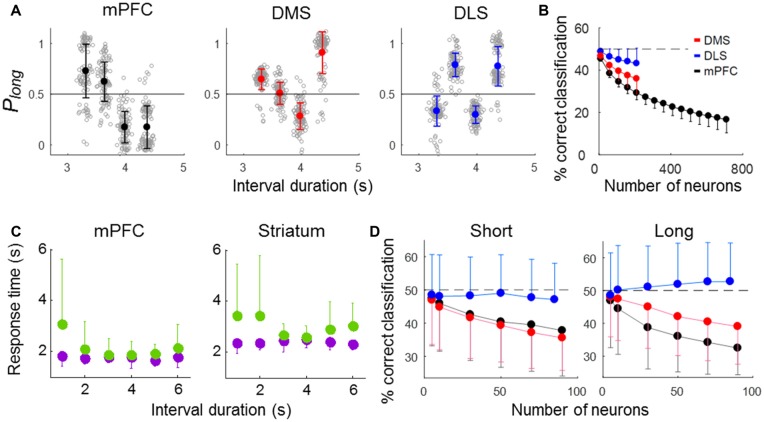
Results of error-trial analysis. **(A,B)** Classification of sample-interval length based on neuronal ensemble activity in error trials. Note that a linear discriminant function was generated using only correct trials. **(A)** Classification of sample-interval length based on all recorded units pooled across sessions. Neural classification was performed using 200 neurons that were selected randomly from each simultaneously recorded ensemble, and this was repeated 100 times. The same format as in Figure 5B. **(B)** Results of a neuron dropping analysis. The same format as in Figure 5C. **(C)** Mean reaction times in correct (purple) and error (green) trials for each sample interval duration. **(D)** Neural classification of sample-interval length for short and long reaction-time error trials (see text). Note that length classification and prediction of the animal’s duration judgment are inversely related in error trials. Error bars, SD.

We next analyzed neuronal activity in error trials to determine whether striatal neural activity correlates with the animal’s judgment of time. We excluded the shortest and longest samples in this analysis because they had so few error trials (3,018 ms, *n* = 4.04 (SD 2.13); 4,784 ms, *n* = 3.31 (SD 1.92) per session). We then generated discriminant functions using the neuronal ensemble activity during the last 500 ms of each sample interval in correct trials and classified sample intervals into short and long ones using neuronal ensemble activity in error trials. The performance of all three ensembles fell significantly below the level expected by chance (50%; *t*-test, ensemble size = 100 neurons, DMS, *t*_(198)_ = 24.14, *p* < 0.001; DLS, *t*_(198)_ = 9.75, *p* < 0.001; mPFC, *t*_(198)_ = 61.07, *p* < 0.001; ensemble size = 200 neurons, DMS, *t*_(198)_ = 20.18, *p* < 0.001; DLS, *t*_(198)_ = 9.46, *p* < 0.001; mPFC, *t*_(198)_ = 62.13, *p* < 0.001). At equivalent ensemble sizes, the mPFC ensemble performance (% correct classification) was significantly lower than that of the other ensembles, and the performance of the DLS ensemble was significantly higher (one-way ANOVA, ensemble size = 100 neurons, *F*_(2,297)_ = 166.05, *p* < 0.001, Tukey’s HSD *post hoc* test, DMS vs. DLS, *p* < 0.001; DMS vs. mPFC, *p* < 0.001, DLS vs. mPFC, *p* < 0.001; ensemble size = 200 neurons, *F*_(2,297)_ = 133.95, *p* < 0.001, Tukey’s HSD *post hoc* test, DMS vs. DLS, *p* < 0.001; DMS vs. mPFC, *p* < 0.001, DLS vs. mPFC, *p* < 0.001; Figures 7A,B). Note that poor classification of sample interval length means good prediction of the animal’s target choice in error trials. Thus, mPFC neuronal activity was most well correlated with the animal’s judgment of sample duration, and DLS neuronal activity was only weakly correlated with duration judgment.

The animal may have committed an error because of imprecise timing-related neural activity even with properly maintained attention (“misclassification” trials). Alternatively, in some error trials, the animal may have totally lost track of time, such as due to poorly maintained attention, and chosen a target without referring to timing-related neural activity (“uncertainty” trials). Because the animal’s reaction time is expected to be relatively short and long in misclassification and uncertainty trials, respectively, we examined how reaction time (the time between sample interval offset and the animal’s arrival at a target) varied as a function of sample interval duration in error trials. The reaction time was relatively short in error trials with “difficult” samples intervals (i.e., those close to the classification boundary; 3,629 and 3,979 ms) and relatively long in error trials with “easy” sample intervals (the shortest and longest ones; 3,310 and 4,363 ms; one-way-ANOVA, mPFC, *F*_(5,267)_ = 6.09; *p* < 0.001; striatum, *F*_(5,414)_ = 4.68; *p* < 0.001; Figure 7C). This is likely due to a high proportion of misclassification for difficult sample intervals. To further explore the relationship between error-trial neural activity and the animal’s target choice, we divided all error trials into two groups according to the reaction time (longer or shorter than the mean reaction time in correct trials for each animal group; mPFC, 1,765.1 ms; striatum, 2,412.4 ms) and repeated the same decoding analysis. For the error trials with relative short reaction times, decoding performance was similarly low for the mPFC and DMS ensembles and close to the chance level for the DLS ensemble (one-way ANOVA, ensemble size = 50 neurons, *F*_(2,2997)_ = 189.30, *p* < 0.001, Tukey’s HSD *post hoc* test, DMS vs. DLS, *p* < 0.001; DMS vs. mPFC, *p* = 0.114, DLS vs. mPFC, *p* < 0.001). For the error trials with relatively long reaction times, the performance of the mPFC ensemble showed the lowest decoding performance, followed by the DMS and then the DLS ensembles (one-way ANOVA, ensemble size = 50 neurons, *F*_(2,2997)_ = 462.18, *p* < 0.001, Tukey’s HSD *post hoc* test, DMS vs. DLS, *p* < 0.001; DMS vs. mPFC, *p* < 0.001, DLS vs. mPFC, *p* < 0.001; Figure 7D). Thus, the mPFC neuronal ensemble activity was well correlated with the animal’s duration judgment for all types of error trials, the DMS ensemble activity was well correlated with the animal’s duration judgment for only short reaction-time error trials, and the DLS neuronal ensemble activity was poorly correlated with the animal’s duration judgment for all types of error trials.

### Temporal Precision as a Function of Time

**Figure 8:**
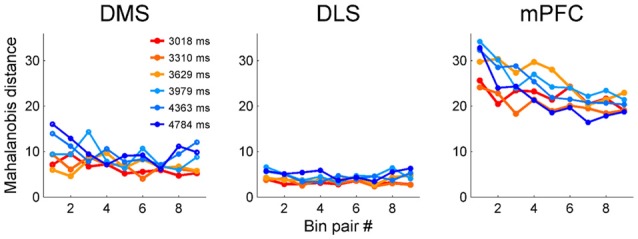
Precision of temporal discrimination as a function of time. We divided sample duration into 10 equal bins after excluding the first 500 ms to minimize the influence of sensory-related neural activity. Then, we calculated Mahalanobis distances between adjacent bins (nine pairs total for each sample interval).

We next examined the relationship between temporal discrimination precision and elapsed time. To avoid sensory cue-related neural activity artifacts, we excluded the first 500 ms of each sample interval from the analysis. We then divided each sample interval into 10 equal bins (the duration of each bin is 10% of a given sample duration) and calculated Mahalanobis distances between adjacent bins based on the neuronal ensemble activity in each bin. The Mahalanobis distance measures how far apart two groups of vectors are considering their centers and variances (McCune et al., 2002). To make it possible to compare the three brain regions, we calculated the Mahalanobis distances after matching ensemble size in each region to that of the smallest ensemble (i.e., DMS, *n* = 311 neurons). We accomplished this by randomly deleting 118 DLS MSNs and 471 mPFC neurons. We repeated this procedure 100 times and calculated the mean Mahalanobis distances for the DLS and mPFC neuronal ensembles. The Mahalanobis distances for the mPFC neuronal ensembles tended to decrease over time (Figure 8); the slopes of the linear regression curves were significantly different from zero for all sample intervals (*t*-test, *t*_(7)_-values > 7.54, *p*-values < 0.029) except the shortest one (*t*_(7)_ = 3.89, *p* = 0.089). In contrast, for the DMS ensembles, the slope for the shortest sample interval (*t*_(7)_ = 10.77, *p* = 0.013) was significantly different from zero, but the slopes for the other intervals were not (*t*_(7)_-values < 4.41, *p*-values > 0.074). For the DLS ensembles, none of the slopes were significantly different from zero (*t*_(7)_-values < 3.34, *p*-values > 0.110; Figure 8). These results are unlikely to reflect different time interval discrimination behavior between the two groups of animals. We found no significant difference in behavior between the two groups (striatum vs. mPFC animals, *t*-test, performance, 79.0 (SD 1.4) vs. 80.0% (SD 4.6) correct choices, *t*_(4)_ = −1.11, *p* = 0.330; parameters of logistic regression analysis (eq. 1), slope, 2.83 (SD 0.23) vs. 3.01 (SD 0.10), *t*_(4)_ = −1.63, *p* = 0.244; intercept, −10.86 (SD 0.78) vs. −11.47 (SD 0.36), *t*_(4)_ = 1.60, *p* = 0.251; *R*^2^-values, 0.961 (SD 0.004) vs. 0.963 (SD 0.009), *t*_(4)_ = −0.38, *p* = 0.739).

### Linear vs. Logarithmic Activity Profile

We showed previously that mPFC neuronal activity profiles are better described by logarithmic than linear functions (Kim et al., 2013). This may explain why the precision of neural decoding of time progressively decreases over time. For comparison’s sake, we asked how well linear vs. logarithmic time scales explain the activity of individual striatal neurons. As in our previous study (Kim et al., 2013), we divided each sample interval into 10 equal bins and used a linear regression model (eq. 2) to examine the dependence of trial-by-trial neuronal activity within each bin on linear or log-transformed time. For the mPFC neurons, we found that the *R*^2^ values determined using the logarithmic model were significantly larger than those determined with the linear model for all sample interval durations (Table 1). In contrast, for the DLS and DMS neurons, we found no significant difference between the *R*^2^ values determined using the logarithmic or linear models for any sample interval duration (Table 1). Because most DMS and DLS MSNs show phasic activity, we repeated this analysis using only the neurons with relatively long activity profiles (half activity duration >400 ms, DMS, *n* = 116; DLS, *n* = 88; mPFC, *n* = 470). This analysis yielded similar results (Table 1). Thus, in contrast with mPFC neurons, there is no significant difference between the ability of logarithmic or linear models to account for the activity of DMS or DLS neurons.

**Table 1:** Comparison of *R*^2^ values for linear vs. logarithmic functions.

DMS	3,018 ms	3,310 ms	3,629 ms	3,979 ms	4,363 ms	4,784 ms
*R*^2^ of linear function	0.115 (0.100)	0.123 (0.118)	0.150 (0.134)	0.145 (0.128)	0.135 (0.123)	0.124 (0.111)
*R*^2^ of log function	0.116 (0.106)	0.124 (0.126)	0.152 (0.146)	0.145 (0.141)	0.136 (0.134)	0.125 (0.116)
*t*-value	−0.371	−0.382	−0789	−0256	−0.443	−0.301
*p*-value	0.711	0.703	0.431	0.798	0.658	0.763
DLS	3,018 ms	3,310 ms	3,629 ms	3,979 ms	4,363 ms	4,979 ms
*R*^2^ of linear function	0.131 (0.135)	0.128 (0.133)	0.141 (0.128)	0.155 (0.151)	0.130 (0.134)	0.111 (0.107)
*R*^2^ of log function	0.130 (0.134)	0.127 (0.136)	0.142 (0.137)	0.156 (0.153)	0.133 (0.143)	0.110 (0.112)
*t*-value	0.405	0.245	−0.596	−0275	−1.307	0.737
*p*-value	0.686	0.807	0.552	0.783	0.192	0.461
mPFC	3,018 ms	3,310 ms	3,629 ms	3,979 ms	4,363 ms	4,979 ms
*R*^2^ of linear function	0.092 (0.102)	0.103 (0.113)	0.110 (0.112)	0.111 (0.113)	0.108 (0.114)	0.108 (0.113)
*R*^2^ of log function	0.096 (0.109)	0.107 (0.119)	0.113 (0.118)	0.114 (0.122)	0.110 (0.120)	0.110 (0.119)
*t*-value	−4.890	−4.248	−3.461	−2.997	−2.590	−1.988
*p*-value	<0.001	<0.001	<0.001	0.003	0.010	0.047
DMS	3,018 ms	3,310 ms	3,629 ms	3,979 ms	4,363 ms	4,784 ms
*R*^2^ of linear function	0.120 (0.112)	0.133 (0.125)	0.154 (0.139)	0.143 (0.126)	0.131 (0.121)	0.122 (0.109)
*R*^2^ of log function	0.124 (0.126)	0.136 (0.135)	0.160 (0.159)	0.147 (0.147)	0.136 (0.138)	0.125 (0.122)
*t*-value	−1.052	−0.830	−1.379	−1.084	−1.286	−0.865
*p*-value	0.295	0.408	0.171	0.280	0.201	0.389
DLS	3,018 ms	3,310 ms	3,629 ms	3,979 ms	4,363 ms	4,979 ms
*R*^2^ of linear function	0.138 (0.144)	0.144 (0.161)	0.133 (0.131)	0.162 (0.172)	0.113 (0.110)	0.102 (0.108)
*R*^2^ of log function	0.143 (0.144)	0.149 (0.165)	0.134 (0.135)	0.162 (0.170)	0.116 (0.124)	0.100 (0.111)
*t*-value	−0.972	−1.525	−0.451	0.091	−0.909	0.536
*p*-value	0.334	0.131	0.653	0.928	0.366	0.593
mPFC	3,018 ms	3,310 ms	3,629 ms	3,979 ms	4,363 ms	4979 ms
*R*^2^ of linear function	0.087 (0.096)	0.098 (0.110)	0.104 (0.107)	0.105 (0.108)	0.102 (0.112)	0.100 (0.107)
*R*^2^ of log function	0.091 (0.102)	0.102 (0.117)	0.107 (0.112)	0.108 (0.117)	0.104 (0.116)	0.102 (0.113)
*t*-value	−3.730	−3.019	−2.382	−2.391	−2.044	−1.897
*p*-value	<0.001	0.003	0.018	0.017	0.042	0.058

*R^2^ values of all neurons (top) or only those with activity half-durations >400 ms (bottom, dorsomedial striatum, DMS, n = 116: dorsolateral striatum, DLS, n = 88; medial prefrontal cortex, mPFC = 470) were compared for the regression models containing linear vs. log-transformed time (eq. 2) for each sample interval duration using t-tests. Data are presented as means (SD)*.

## Discussion

The frontal cortex-basal ganglia circuit is strongly implicated in interval timing (Buhusi and Meck, 2005; Meck et al., 2008), but the exact contributions of each circuit component to interval-timing behavior and their underlying neural processes remain unclear. We, therefore, compared neuronal activity patterns in the mPFC, DMS and DLS during identical behavioral tasks. We found that we were able to decode elapsed time from the neuronal ensemble activity recorded from all three regions, meaning all three regions carry substantial temporal information. The neuronal discharge characteristics we recorded from the DMS and DLS during sample interval presentation, however, were distinct from those recorded in the mPFC. DMS and DLS neurons seldom showed the prolonged ramping activity spanning the entire interval that was frequently observed in the mPFC. Instead, most DMS and DLS neurons showed periodic phasic discharges within the sample intervals. In addition, whereas mPFC neuronal activity tended to change logarithmically over time making the neural decoding of elapsed time less precise, this was not the case for DMS or DLS neurons.

Ramping and sequential discharges, which are both found ubiquitously in the brain (Durstewitz and Seamans, 2006; Buzsáki, 2010), are excellent candidate neural processes for conveying temporal information. Both ramping activity and sequential discharges are found in the mPFC as well as striatum (e.g., Kim et al., 2013; Emmons et al., 2017; Tiganj et al., 2017). However, in our task in which the confounding effect of movement-related neural activity is relatively small (Figure 6), activity durations were significantly shorter for striatal than mPFC neurons. Moreover, ramping activity spanning the entire duration of the longest sample interval (4874 ms) was less frequently found in the striatum. Previous studies also have found phasic discharges of striatal neurons under diverse behavioral conditions (e.g., Kimura et al., 1990; Apicella et al., 1991; Lau and Glimcher, 2008). These results raise the possibility that the mPFC and striatum may carry temporal information via distinct neural processes. Why is prolonged ramping observed rarely in the striatum? This may be due to a fundamental difference in the organization of the neural circuits in each region. Cortical circuits mainly consist of excitatory neurons interconnected by recurrent collaterals, whereas the principal striatal neurons (i.e., the MSNs) are inhibitory. Excitatory network activity may drive ramping activity in some PFC neurons (Mongillo et al., 2003). Alternatively, mPFC pyramidal neurons and striatal MSNs may have different channel compositions. In other words, mPFC neurons may exhibit ramping activity because of unique internal channel dynamics (Durstewitz, 2003) that are absent in striatal neurons. Additional studies will be necessary to clarify the mechanism underlying this difference.

There is disagreement about whether the brain represents time on a linear (Gibbon, 1977; Gibbon and Church, 1981; Roberts, 1981; Church and Gibbon, 1982; Gallistel, 1999; Wearden and Jones, 2007) or logarithmic (Church and Deluty, 1977; Staddon and Higa, 1999; Roberts, 2006; Yi, 2009) scale. Some studies have reported linear changes in neural activity over time (e.g., Komura et al., 2001; Machens et al., 2010), but we showed that activity profiles of mPFC neurons are better described by logarithmic than linear functions in the current task (Kim et al., 2013). The logarithmic encoding of time means that as time elapses, its representation becomes progressively less precise. This was indeed the case for mPFC neurons regardless of whether we analyzed the whole population (Kim et al., 2013), analyzed the phasic-firing “time cells” alone (Tiganj et al., 2017), or excluded the putative sensory-related responses (Figure 8). In contrast, when we excluded the putative sensory-related responses (i.e., the first 500 ms) from the analysis, the DMS and DLS showed similar levels of precision in temporal discrimination over time. Also, unlike with the mPFC neurons, logarithmic functions were no better at accounting for the temporal profiles of individual striatal neurons than linear functions. With the caveats that we recorded the striatal and mPFC neurons from different animals and that the results of comparing linear vs. logarithmic functions were equivocal for striatal neurons, our results raise the possibility that different brain areas may represent time on linear and logarithmic scales in parallel.

Our finding that DMS neurons convey more temporal information and predict animal choices better in error trials than DLS neurons is consistent with the anatomical connections between the mPFC and striatum (the mPFC directly projects to the DMS, but not the DLS; McGeorge and Faull, 1989; Voorn et al., 2004; Balleine et al., 2007; Redgrave et al., 2010; Devan et al., 2011) and recent physiological studies that suggest the transfer of temporal information from the PFC to the striatum (Emmons et al., 2017; Wang et al., 2018). Although it is unknown whether other cortical regions (e.g., the sensorimotor cortex) similarly convey temporal information to the DLS, our results suggest the mPFC may be an important source of temporal information for the DMS. Then a question arises as to why we found evidence for logarithmic encoding of time in the mPFC, but not in the striatum. It would be informative in the future to examine the effects of manipulating the mPFC on DMS timing-related neural activity in order to understand whether and how mPFC neural activity contributes to temporal information processing in the DMS (see Emmons et al., 2017; Wang et al., 2018). It is also unclear whether close interactions between the PFC and striatum are necessary for all types of interval timing behavior (Matell and Meck, 2004; Meck et al., 2008; Agostino and Cheng, 2016) or they may work independently to be in charge of controlling different types of interval timing behavior. A recent study has shown that striatal units predict the timing of licking responses better than orbitofrontal cortical units in head-fixed mice performing an appetitive trace conditioning task (Bakhurin et al., 2017). In our study, the mPFC neural activity was most well correlated with the animal’s target choice in all types of error trials. The mPFC and striatum may play more important roles in controlling where to navigate according to duration judgment (as in our task) and when to emit a specific motor response (such as licking), respectively, for example. In general, it is largely unknown how temporal information contained in temporally-changing neural activity in a given brain area contributes to behavioral control. Clearly, further studies will be necessary to clarify the functional roles of timing-related neural activity in different areas of the brain.

## Author Contributions

JK and MWJ designed the study. JK performed the experiments. JK, DK and MWJ analyzed the data. MWJ wrote the article with input from all authors.

## Conflict of Interest Statement

The authors declare that the research was conducted in the absence of any commercial or financial relationships that could be construed as a potential conflict of interest.
